# Anti-hyperplasia Effects of Total Saponins From Phytolaccae Radix in Rats With Mammary Gland Hyperplasia via Inhibition of Proliferation and Induction of Apoptosis

**DOI:** 10.3389/fphar.2018.00467

**Published:** 2018-05-23

**Authors:** Xiaoliang Li, Zhibin Wang, Yu Wang, Yanan Zhang, Xia Lei, Ping Xin, Xin Fu, Ning Gao, Yanping Sun, Yanhong Wang, Bingyou Yang, Qiuhong Wang, Haixue Kuang

**Affiliations:** ^1^Key Laboratory of Chinese Materia Medica (Ministry of Education), Heilongjiang University of Chinese Medicine, Harbin, China; ^2^Science of Chinese Materia Medica, Jiamusi College, Heilongjiang University of Chinese Medicine, Jiamusi, China; ^3^Science of Processing Chinese Materia Medica, School of Traditional Chinese Medicine, Guangdong Pharmaceutical University, Guangzhou, China

**Keywords:** total saponins of Phytolaccae, mammary gland hyperplasia, sex hormones, apoptosis, proliferation

## Abstract

Mammary gland hyperplasia (MGH) is a pathological condition that affects the majority of women at the child-bearing stage. The hormone and endocrinal therapy are typically used to treat MGH. Nevertheless, there are still some certain side effects accompanied with the benefits, which negatively affect the life quality of patients. Therefore, plant-derived agents that are effective against MGH development and with fewer side effects should be developed. The aim of this study was to investigate the protective effects and underlying mechanism of total saponins of Phytolaccae (TSP) against MGH *in vivo*. The results showed that treatment with TSP could significantly correct the disorder of serum sex hormones levels in rats with MGH, and eliminate the formation of MGH. Moreover, TSP significantly protected estrogen and progesterone-induced MGH histological changes, inhibited the swelling of the nipple, and improved the organ coefficient of uterus in rats with MGH. Mechanistically, TSP treatment not only effectively suppressed the mRNA and protein expression of ERα and PR in mammary gland, but also simultaneously up-regulated ERβ expression, and thus blocked sex hormones from interacting with their receptors. TSP treatment markedly suppressed mammary phosphorylation levels of ERK1/2, as well as reduced the mRNA and protein overexpression of VEGF and bFGF in mammary of rats. In addition, TSP treatment substantially down-regulated the expression of Bcl-2 and Ki-67, as well as elevated the expression of Bax. These findings indicated that TSP could potentially be used for effective treatment of MGH.

## Introduction

Nowadays, the disease of MGH, especially for women of child-bearing age, is one of the most common and frequently occurring disease and affects people in increasing numbers, which is classified into the category of “Rupi” in Chinese medicine (Zheng et al., 2013; Chen et al., 2015). Without getting any effective control, MGH shows a severe cancerous tendency, and may have been confused and covered with early breast cancer (Wang L. et al., 2011; Wang Z.C. et al., 2011). According to the modern medicine, the occurrence of MGH is closely related to endocrine disorder, and prescription of steroids (Lin et al., 2015) and surgery are the main treatment measures employed. Nevertheless, the potentially severe side effects and complications also emerge and seriously affect the life quality of patients (Henry, 2014), which is therefore prompt us to explore novel, alternative and effective treatments against the disease. Studies have proved that traditional Chinese medicine have the protective effect against MGH via possible regulated mechanism (Li et al., 2017).

Phytolaccae Radix, including *Phytolacca acinosa* and P. Americana, is recorded in Chinese Pharmacopeia (2015 edition) for its important medicinal value. The chemical constituents of Phytolaccae Radix mainly consist of triterpenoid saponins, flavone, phenolic acid, sterol, and polysaccharides. There have been an increasing number of studies focusing on its bio-activities, including diuretic, antibacterial, antiviral, anti-inflammatory anti-tumor and anti-MGH (Wang et al., 2014). It has a long history of use in the treatment of MGH. Patients with MGH achieved good therapeutic effect after taking the tablets which were made by the fresh Phytolaccae Radix (Tian et al., 1985).

The objective of the present study is to investigate the effects and potential mechanisms of TSP to protect against MGH in rats induced by estrogen combined with progestogen. This is first study on anti-MGH effect of TSP *in vivo*.

## Materials and Methods

### Chemicals and Regents

Estradiol (E2) Benzoate Injection and Progesterone (P) Injection was supplied by Ningbo SANSHENG Pharmaceutical Co., Ltd. (Ningbo, Zhejiang, China). Smoothing and moisturizing hair removal was obtained from Watsons. E2, P, luteinizing hormone (LH), follicle stimulating hormone (FSH), prolactin (PRL), and testosterone (T) and T ELISA kits were purchased from Nanjing Jiancheng Bioengineering Institute (Nanjing, Jiangsu, China). The primary antibodies for VEGF, bFGF, p-ERK1/2, ERK1/2, Estrogen Receptor α, Estrogen Receptor β, ki-67, Bax, Bcl-2, GAPDH, and β-actin as well as all of the secondary antibodies were purchased from Cell Signaling Technology (Danvers, MA, United States).

### Total Saponins Extraction and Isolation

The preparation methods of TSP were coincided with the previous study (Wang, 2015). Briefly, the dried Phytolaccae Radix were powdered using an oscillating high speed universal grinder. The power was extracted thrice with water for 1.5 h at each time. The solution was filtered, concentrated, subjected to a HPD100 macroporous adsorption resin column, and then eluted successively with deionized water, 10, 30, and 70% aqueous ethanol. Subsequently, the solutions eluted by water, 10 and 30% aqueous ethanol were discarded. The remaining 70% aqueous ethanol solution was collected and concentrated by a rotary evaporator, and then the concentrated extract of the total saponins was dried in a lyophilizer.

### Ultra-Performance Liquid Chromatography-Q/Time-of-Flight Mass Spectrometry (UPLC-Q/TOF-MS) Analysis

Chromatographic analysis was carried out using an Acquity UPLC system (Waters, Milford, MA, United States) with a 3 μL injection volume. Next, Tandem MS was performed using a Q-TOF mass spectrometer (Waters) with an Electron Spray Ionization. The results were analyzed using MassLynx v4.1 and UNIFI v1.8 (Waters).

### Animal Experiments

The virgin female Wistar rats weighing 180–220 g (license number: SCXK2015-0001) were commercially obtained from Liaoning Changsheng Biotechnology Co., Ltd. The rats were housed at a controlled room (temperature 23 ± 1 degree Celsius and humidity 60 ± 5%) under a 12 h light-dark cycle, and fed with standard diet and water *ad libitum*. They were acclimated under climate-controlled conditions for 7 days before the experiments began.

After acclimatization to the laboratory conditions, the rats were randomly divided into normal group (NO group) and MGH group (MGH group). The rats in MGH group were injected estrogen (0.5 mg/kg/d) into the muscle of medial side of hind leg for 25 days, and followed with progestogen (5 mg/kg/d) for another 5 days to induce animal model of MGH, meanwhile, the NO group were administered with normal saline intramuscularly for 30 days (Wang L. et al., 2011). From the 31st day, the rats with MGH were further randomly separated into six groups of ten individuals and treated as follows: (1) the vehicle control group (an equal volume of normal saline, i. g.); (2) the MGH model group (an equal volume of normal saline, i. g.); (3) Positive control group (tamoxifen, 1.5 mg/kg bwt, i. g.); (4) total saponins (7.5 mg/kg bwt, i. g.); (5) total saponins (15 mg/kg bwt, i. g.); (6) total saponins (30 mg/kg bwt, i. g.). All drugs were dissolved in distilled water before use and treatments were administered by gastric gavage at 10 ml/kg bodyweight for a total of 30 days continuously. Nipple diameter of rats were measured at the predetermined time during the experiment. At the end of the experimental period, all rats were fasted overnight and anesthetized using 20% urethane 1.0 g/kg body weight injected intraperitoneally. Blood samples were obtained via abdominal aorta, the serum was separated by centrifugation (3000 rpm for 10 min at 4 degree Celsius), and then stored at minus 80 degree Celsius for hormone assays. The tissues of mammary gland were fixed in 10% neutral buffered formalin or kept at minus 80 degree Celsius for subsequent analysis. This study was approved by the Animal Care and Use Committee of Heilongjiang University of Chinese Medicine.

### Determination of Nipple Diameter and Organ Coefficients

The diameter of the rat’s nipple was measured with vernier caliper on the 1st day, 30th day, 45th day, and the last day of the experiment. The uterus and ovary were completely separated and weighed at the end of the experiment. Then, the organ coefficients were calculated as uterus or ovary weight divided by body weight.

### Biochemical Analysis and Enzyme-Linked Immunosorbent Assay (ELISA)

The concentrations of E2, P, LH, FSH, PRL, and T in serum were measured by commercial detection kits according to the procedures recommended by the manufacturer (Nanjing Jiancheng Bioengineering Institute, Nanjing, China).

### Real Time PCR Analysis

Real-time PCR was performed to determine gene expression of ERα, ERβ, PR, VEGF, bFGF, Bcl-2, and Bax. Total RNA was extracted from the tissues of mammary gland with Trizol (Invitrogen) following the manufacturer’s protocol. The cDNA was synthesized from 1 μg of total RNA using the Reverse Transcription Reagent Kit (TaKaRa, Shiga, Japan) in accordance with instruction manual. The sequences of the primers are listed in **Table 1**. The following thermocycling protocol was used: at 95 degree Celsius for 30 s, 40 cycles at 95 degree Celsius for 5 s and at 60 degree Celsius for 40 s. The relative quantities of the candidate genes and GAPDH mRNA were calculated by comparative CT method. The experiments were repeated for three times. Samples in each experiment were in triplicate.

**Table 1:** Primers used in the qRT-PCR study.

Genes	Forward (5′-3′)	Reverse (5′-3′)
ERa	TTGCTCCTAACTTGCTCTTGG	TGCGGAATCGACTTGACG
ERβ	ATTTTCGCTCCCGACCTC	TAACTCACGGAACCTTGACG
PR	CCCAGTTCACAACGCTTCTAT	CTGAGACAAAATGACACACCACA
VEGF	CAAAGCCAGCACATAGGAGAGAT	TTTTTGCAGGAACATTTACACGTC
bFGF	GAAGAGCGACCCACACGTCAAAC	TCCCTTGATGGACACAACTCCTCTC
GAPDH	TTCCTACCCCCAATGTATCCG	CCACCCTGTTGCTGTAGCCATA


### Western Blot Analysis

The protein samples from the tissues of mammary gland were isolated using a protein extraction kit, and the protein concentrations were determined using a BCA protein assay kit. Equal amounts of proteins (50 μg) were separated by 10% SDS–PAGE and then transferred onto polyvinylidene difluoride (PVDF) membranes. After blocking with 5% nonfat dry milk in Tris-buffered saline Tween (TBS-T) for 2 h at room temperature, the membranes were individually incubated with the primary antibodies overnight at 4 degree Celsius. Following incubation with HRP-conjugated secondary antibody for 2 h at room temperature, the protein bands were visualized by ECL Prime Western Blotting Detection Reagent (Bio-Rad, United States). Chemiluminescent signals were detected and analyzed with the ChemiDoc XRS imaging system (Bio-Rad, United States). The intensity of protein bands was quantified using the Image J Software. The experiments were repeated for three times. Samples in each experiment were in triplicate.

### Histological Analysis

The tissues of mammary gland were obtained for histopathological examination. In detail, the specimens of the tissues of mammary gland were fixed in 10% neutral buffered formalin for 48 h. After processed in a series of graded ethanol and dimethyl benzene, the tissues were embedded in paraffin, cut into 4 μm thick sections, and then stained with hematoxylin and eosin (H&E). Finally, we observed pathological changes in the tissues of mammary gland by using an SZX10 microscope (Olympus Corp., Tokyo, Japan).

### Immunohistochemistry

Paraffin-embedded sections (4 μm) were dewaxed in xylene, sequentially rehydrated in alcohol and incubated in 3% H_2_O_2_ for 20 min. The sections were heated twice in a microwave oven for 5 min in 0.01 M citrate buffer at pH 6.0 for antigen retrieval and followed by overnight incubation at 4 degree Celsius with the primary antibodies: Ki-67 at 1:100, Bcl-2 at 1:50 and Bax at 1:50. The sections were washed and incubated with the HRP-conjugated secondary antibody for 30 min at 37 degree Celsius. After staining with DAB, the tissue slides were counterstained with hematoxylin, dehydrated through a graded ethanol series, and sealed with neutral gum in the end. The sections were captured under microscope with a camera (Olympus, Tokyo, Japan).

### Statistical Analysis

Statistical analysis was performed with SPSS 18.0 software. All data were presented as mean ± SE. Comparisons of numerical data between two groups were calculated by Student *t*-tests. Differences in mean values of various groups were analyzed by ANOVA. Difference with *P*-value < 0.05 was considered as statistically significant.

## Results

### Identification of the Chemical Composition of TSP by UPLC-Q/TOF-MS

**FIGURE 1:**
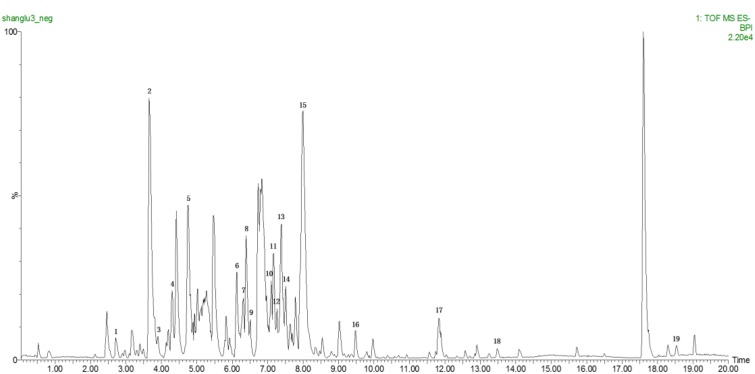
Base peak ion (BPI) chromatogram of TSP in negative ion mode determined by ultra-performance liquid chromatography-Q/-time-of-flight-mass spectrometry (UPLC-Q/TOF-MS).

**Figure 1** shows the base peak ion (BPI) chromatogram of TSP. A total of 19 compounds were initially identified based on retention time, molecular ions, major fragment ions, and previously published articles and online databases. The identified compounds were mainly classified as triterpene glycoside. The details of the identified compounds are listed in **Table 2**.

**Table 2:** Chemical composition of TSP determined by UPLC-Q/TOF-MS.

Peak	Component name	RT (min)	Molecular formula	Neutral mass (Da)	Adducts
1	Esculentoside N	2.72	C_54_H_86_O_26_	1150.54073	+HCOO, -H
2	Esculentoside K	3.75	C_54_H_86_O_25_	1134.54582	+HCOO, -H
3	Phytolaccoside B	3.91	C_36_H_56_O_11_	664.38226	+H, +Na, +NH4
4	Esculentoside I	4.31	C_49_H_78_O_22_	1018.49847	-H
5	Esculentoside F	4.76	C_41_H_64_O_16_	812.41944	+HCOO, -H
6	Esculentoside G	6.10	C_48_H_74_O_21_	988.48791	+HCOO
7	Esculentoside H	6.28	C_48_H_76_O_21_	988.48791	+HCOO, -H
8	Esculentoside L	6.31	C_48_H_76_O_20_	972.49299	+HCOO, -H
9	Phytolaccoside E	6.51	C_42_H_66_O_16_	826.43509	+HCOO, -H
10	Phytolaccoside F	7.17	C_48_H_76_O_19_	956.49808	+HCOO, -H
11	Esculentoside D	7.27	C_37_H_58_O_12_	694.39283	+HCOO, -H
12	Phytolaccoside D	7.38	C_42_H_66_O_15_	810.44017	+HCOO, -H
13	2-hydroxyl esculentic acid	7.44	C_30_H_46_O_7_	518.32435	-H, +HCOO
14	Esculentoside A	7.51	C_42_H_66_O_16_	826.43509	+HCOO, -H
15	Esculentoside B	7.99	C_36_H_56_O_11_	664.38226	+HCOO, -H
16	Esculentoside C	9.48	C_42_H_66_O_15_	810.44017	+HCOO, -H
17	Phytolaccagenin	11.84	C_31_H_48_O_7_	532.34000	+NH4, +Na, -e
18	Esculentoside E	13.49	C_35_H_54_O_11_	650.36661	-H
19	Phytolaccagenic acid	18.54	C_31_H_48_O_6_	516.34509	-H


### TSP Improved the Nipple Diameter of Rats With MGH

**FIGURE 2:**
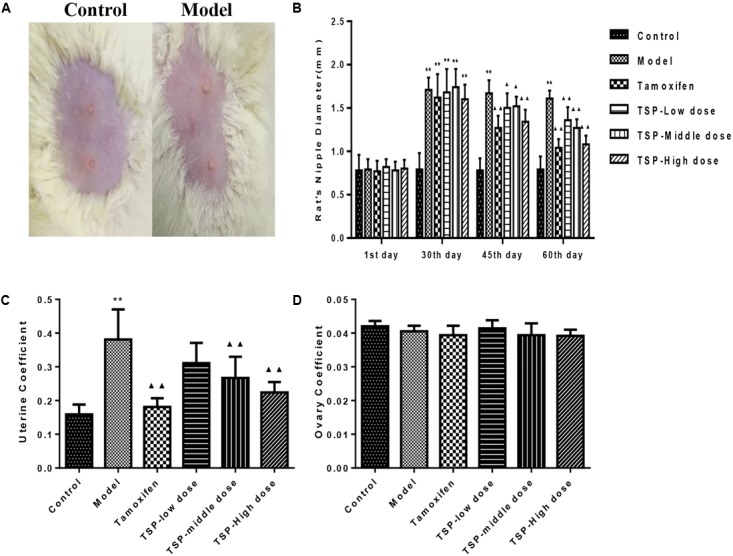
Effects of TSP on the nipple diameters and organ coefficients of uterus and ovary index in rats with MGH. **(A)** Representative photograph of rat breasts. **(B)** Comparison of nipples diameter in rats. **(C)** Coefficient of uterus. **(D)** Coefficient of ovary in rats. Data were shown as mean ± SE (*n* = 10), ^∗∗^*P* < 0.01 vs. control group; ^▴^*P* < 0.05 and ^▴▴^*P* < 0.01 vs. model group.

As shown in **Figure 2A**, the nipples of MGH model rats were become swelling and larger than those of the normal control. The nipple diameters (Left 2 and Right 2) were markedly increased by intramuscular injection of estrogen and progestin compared with normal control group. After 2 weeks’ administration of TSP, the nipple diameters were obviously decreased, and further reduced at the end of the treatment. The change in nipple diameters over time is shown in **Figure 2B**.

### Effects of TSP on Organ Coefficients of Uterus and Ovary in Rats With MGH

To further investigate the effects of TSP on MGH, the organ coefficients of uterus and ovary in rats of each group were obtained. Results showed that compared with normal control group, the organ coefficient of uterus in MGH model group were obviously increased. However, the organ coefficient of uterus was markedly decreased by TSP-Middle and -High dose treatment as compared with MGH model group. There were no outstanding differences in the organ coefficients of the ovary between all the groups (**Figure 2**).

### Effect of TSP on Pathomorphology of Mammary Gland Tissue in MGH Model Rats

**FIGURE 3:**
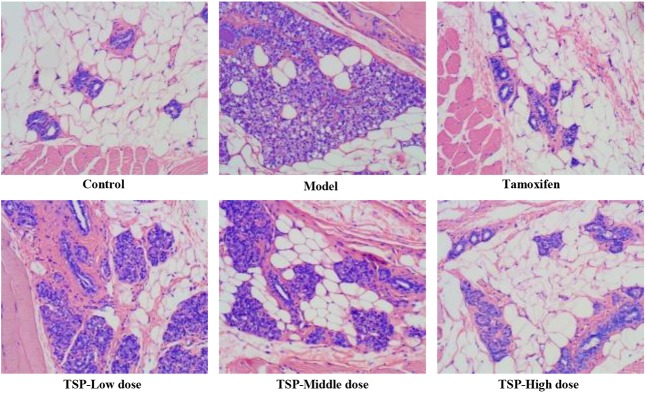
Morphology of mammary gland tissue was observed by microscope (original magnification, 100×).

To verify the effectiveness of TSP in mitigating MGH, H&E staining was conducted and histological variation was observed. As can be seen from **Figure 3**, there were no histopathological alterations in the mammary gland of the control group rats. In contrast in the model group, mammary epithelial cell tissue had significantly proliferative lesions, including hyperplasia in most lobules, increased numbers of acini and ducts, and thickened glandular epithelium. Administration of tamoxifen and TSP-High dose for four consecutive weeks significantly suppressed these typical histological patterns. The proliferative degree of mammary lobules and the number of acini and ducts markedly decreased. While daily treatment of TSP-Low and -Middle dose for 4 weeks, compared to the model group, were also capable to decrease the lumen area and numbers of mammary acini and ducts in different degree. These results illustrated that TSP had therapeutic effect on the rats with MGH induced by estrogen and progestogen.

### TSP Ameliorated the Levels of Serum Sex Hormones in Rats With MGH

**FIGURE 4:**
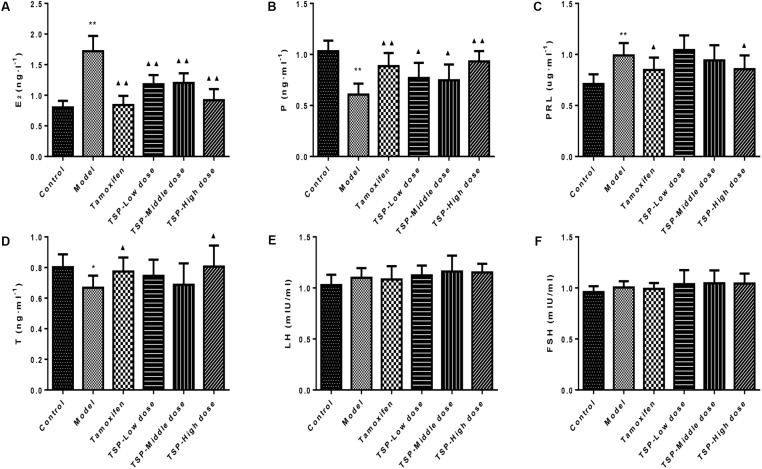
Effects of TSP on sex hormones levels in rat’s serum. **(A)** E2, **(B)** P, **(C)** PRL, **(D)** T, **(E)** LH, and **(F)** FSH in rat’s serum. Values are expressed as mean ± SE (*n* = 10). ^∗^*P* < 0.05, and ^∗∗^*P* < 0.01 vs. control group; ^▴^*P <* 0.05 and ^▴▴^*P <* 0.01 vs. model group.

After injection of estrogen and progestogen, as shown in **Figure 4**, E2 and PRL contents were obviously increased (*P* < 0.01 for both cases), while P (*P* < 0.01) and T (*P* < 0.05) contents were notably decreased when compared to the control group, and most noticeably, there was no significant changes in LH and FSH contents in each group. However, the administration of TSP at different concentrations decreased the level of E2 and increased the level of P, but only TSP at a high dose reduced the contents of PRL and elevated contents of T. Our results suggested that TSP could adjust the serum E2, P, PRL, and T level of rats with MGH to play anti-MGH function.

### TSP Modulated the mRNA and Protein Expression of ERα, ERβ, and PR in Mammary Gland of Rats With MGH

**FIGURE 5:**
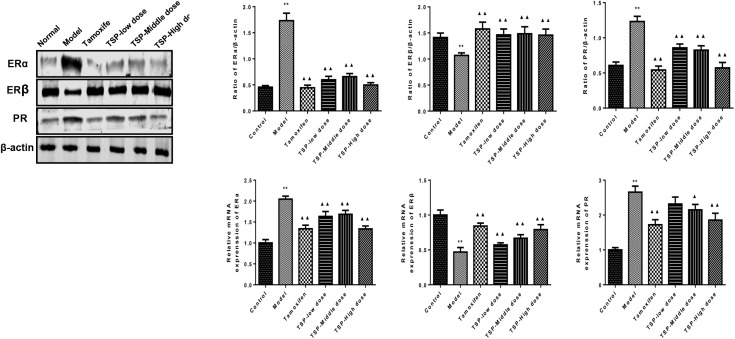
Effects of TSP on the mRNA and protein expression of ERα, ERβ, and PR in mammary gland of rats with MGH. Data were shown as mean ± SE from three independent experiments, ^∗^*P* < 0.05, and ^∗∗^*P* < 0.01 vs. control group; ^▴^*P <* 0.05 and ^▴▴^*P <* 0.01 vs. model group.

ER is an important nuclear transcription factor activated by ligand. As two kinds of subtypes, ERα and ERβ mediate estrogen and make it work. Meanwhile, PR is depending on the existence of ER, which is regulated and controlled by estrogen (Guo et al., 2017). To investigate the effect of TSP on ERα, ERβ and PR, the levels of related protein and mRNA were detected. As shown in **Figure 5**, The protein and mRNA expressions of ERα and PR were elevated in comparison with control group, while protein and mRNA expressions of ERβ were reduced in mammary gland of rats with MGH. However, TSP, at any dose, showed significant inhibitory effects on the protein and mRNA expressions of ERα and remarkable promoting effects on the protein and mRNA expressions of ERβ. Moreover, TSP treatment also exhibited the decrease in PR protein expressions in mammary gland of rats with MGH. Compared with that of model group, the mRNA levels of PR in mammary gland of rats with MGH were notably decreased after the administration of these MGH rats with TSP middle-(*P* < 0.05) and high-doses (*P* < 0.01).

### TSP Reduced the mRNA and Protein Expression of VEGF and bFGF, and Inhibited the Phosphorylation of ERK1/2 in Mammary Gland of Rats With MGH

**FIGURE 6:**
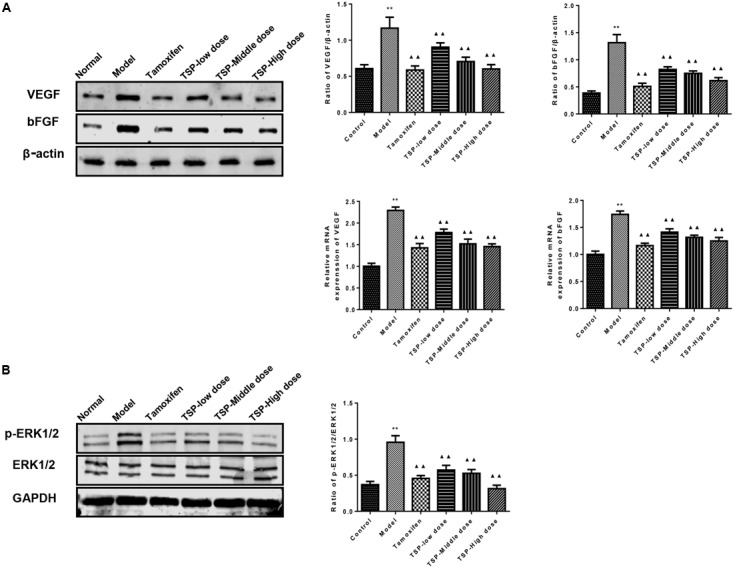
Effects of TSP on the mRNA and protein expression of VEGF and bFGF and the phosphorylation of ERK1/2 in mammary gland of rats with MGH. **(A)** The mRNA and protein expression of VEGF and bFGF in different groups were determined respectively by reverse transcription quantitative polymerase chain reaction and western blot. **(B)** ERK1/2 phosphorylation in different groups were detected by western blot. Representative western blots images are displayed. Data were presented as mean ± SE from three independent experiments, ^∗^*P* < 0.05, and ^∗∗^*P* < 0.01 vs. control group; ^▴^*P <* 0.05 and ^▴▴^*P* < 0.01 vs. model group.

The effects of TSP on the expression of VEGF and bFGF were determined by qPCR and western blot in mammary gland. The results showed that the mRNA and protein expressions of VEGF and bFGF were increased in MGH rats induced by estrogen and progesterone. TSP treatment inhibited the protein and mRNA expressions of VEGF and bFGF in a dose-dependent manner in mammary gland. (*P* < 0.01, **Figure 6A**). The phospho-ERK1/2 levels were also altered after estrogen and progesterone induction, and the application of TSP reduced ERK1/2 activation in a dose-dependent manner in mammary gland. (*P* < 0.01, **Figure 6B**).

### TSP Induced Apoptosis in Rats With MGH

**FIGURE 7:**
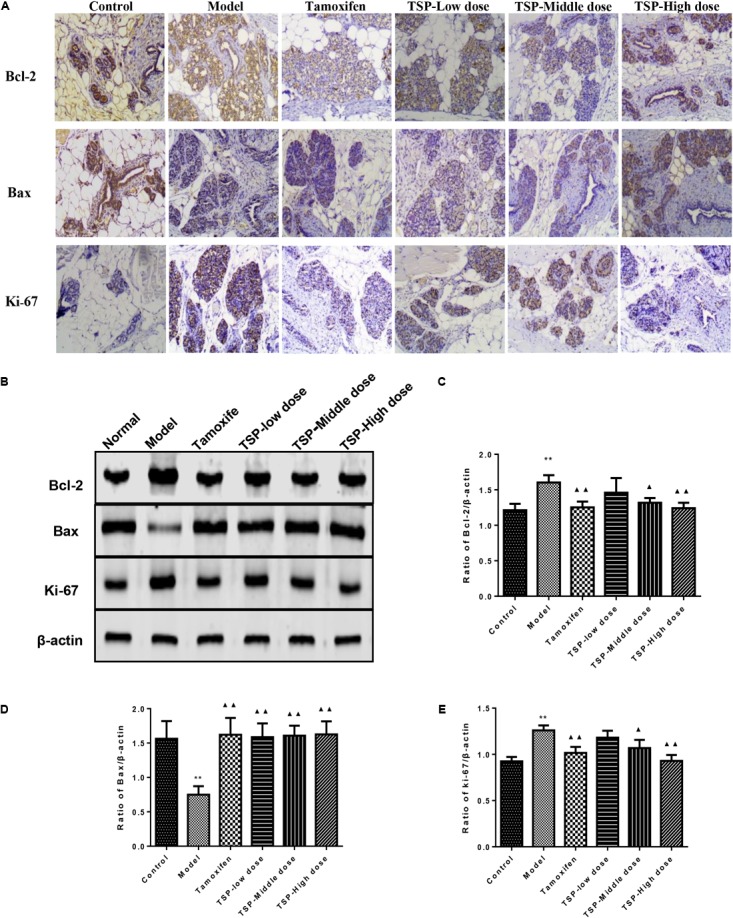
Effects of TSP on level of Bcl-2, Bax and ki-67 in mammary gland of rats with MGH. **(A)** Immunohistochemistry findings of Bcl-2, Bax and ki-67 in each group. Tissue sections from mammary gland in each group were stained with anti-Bcl-2, anti-Bax, and anti-ki-67. Original magnification 100×. **(B–E)** The expression was detected by western-blot. Representative western blots images are displayed. Data were presented as mean ± SE from three independent experiments, ^∗^*P* < 0.05, and ^∗∗^*P* < 0.01 vs. control group; ^▴^*P* < 0.05 and ^▴▴^*P* < 0.01 vs. model group.

The expression of Bcl-2, Bax, and ki-67 in mammary gland were detected by immunohistochemistry and western blotting, so that the action mechanism of TSP in rats with MGH could be investigated. The results could be seen in **Figures 7A–E**, which showed that TSP at a middle-(*P* < 0.05) and high-doses (*P* < 0.01) significantly down-regulated the levels of Bcl-2 and ki-67 compared with those in the model group, but had no effect at low dose. In addition, treatment of MGH rats with TSP also up-regulated level of Bax (*P* < 0.01) in MGH rat’s mammary gland. These findings indicated that TSP could significantly promote apoptosis in rats with MGH.

## Discussion

Modern medicine believes that the breast is the target organ of the sex hormones, and the main cause leading to the MGH is the disorder of hormones metabolism in the body. The increased E2 in the absolute or relative terms and a persistent lack of P make the breast excessive hyperplasia and incomplete restoration (Schindler, 2011). PRL which is produced by eosinophilic cells in the distal part of the adenohypophysis promotes the growth and development of the mammary gland under the combined action of E2 and P. T is mainly secreted by the ovary and adrenal cortex in women. A small amount of T promotes the development of mammary glands. On the contrary, a large number of T plays an inhibitory effect (Han et al., 2015). In the present study, the continuous stimulation of exogenous hormones eventually leads to abnormal secretion of serum sex hormones in female rats. The treatment of TSP for consecutive 30 days could effectively regulate the levels of E2, P, PRL, and T hormones in serum of rats with MGH.

The effect of sex hormones on the body is mediated through the specific binding of the receptor. Estrogen action in target tissues is achieved by binding to specific nuclear receptors, estrogen receptors α and β (Higa and Fell, 2013). These receptors play a critical role in the regulation of cell proliferation and differentiation in the mammary glands, uterus, and other tissues (Jonsson et al., 2014). Activation of ERα supports cell proliferation and differentiation in the breast and other tissues. And recent studies suggest that high ERβ content can down-regulate the expression of ERα (Stope et al., 2010; Mehta et al., 2014). PR is regulated by estrogen, and its synthesis in normal mammary cells requires the combination of estrogen and estrogen receptor (Savouret et al., 1991; Lian et al., 2017). The increase of ERα and PR will definitely increase the sensitivity of breast epithelial to estrogen, and induce the occurrence of MGH. Here, we found that TSP effectively suppressed the mRNA and protein expression of ERα and PR, and simultaneously up-regulated ERβ expression in mammary gland of rats.

VEGF is a specific mitogen and angiogenic factor which stimulates endothelial cell growth and proliferation, promotes the generation of new blood vessels and regulates vascular permeability (Queiroga et al., 2010; Anadol et al., 2017). bFGF can promote mitosis, especially for the proliferation and differentiation of mesoderm cells. Although VEGF and bFGF have different mechanisms for promoting angiogenesis, there is a good synergy between them. As the degree of MGH increases, the activity of new blood vessels increased in tissues (Tao et al., 2017). ERK is an important member of mitogen activated protein kinase (mitogen activated protein kinase, MAPK) family. The combination of growth factors and hormones with neurotransmitter receptors will lead to the phosphorylation of ERK, promote the activated factor into the nucleus, and then participate in cell proliferation and differentiation (Feng et al., 2015; An et al., 2017). One study showed that estrogen activated the ERK1/2 pathway and increased the expression of VEGF. The higher the expression of VEGF, the more abundant the new blood vessel were produced and hence hyperplasia will be aggravated (Shan et al., 2013; Lin et al., 2015). These are consistent with the phenomena in our present study. 30 continuous days’ administration of TSP markedly attenuated the MGH rat induced by estrogen combined with progestogen through suppressing mammary phosphorylation levels of ERK1/2, as well as reducing the mRNA and protein expression of VEGF and bFGF.

Apoptosis, also known as programmed cell death, refers to an active and ordered form of cell death, which are mainly genetically controlled (Zaman et al., 2014; Wei et al., 2017). The Bcl-2 protein family includes key regulatory factors for apoptosis, such as anti-apoptotic protein Bcl-2 and pro-apoptotic protein Bax, both of which are generally considered as important molecular proteins for apoptosis (Zhang and Saghatelian, 2013; Zhao et al., 2017). Ki-67 was found by Gerdes et al. (1984), and increased in mitotic stage, especially in the M phase of tumor active stage, and then decreased rapidly after mitotic cycle (Ding et al., 2004). With the development of science and technology, there are more and more researches on the influence of Chinese medicine treatment on the biological behavior of Ki-67. Many studies have found that Ki-67 is highly expressed in the tissue of MGH (Chen et al., 2014; Zhou and Wu, 2016). In concert with these findings, we found in this study that TSP substantially down-regulated the expression of Bcl-2 and Ki-67, as well as elevated the expression of Bax.

## Conclusion

The whole results confirmed that TSP markedly attenuated MGH through rectifying the disorder of sex hormone, regulating the expression of hormone receptor, inhibiting the proliferation of mammary tissue cells, promoting apoptosis and inhibiting angiogenesis. Based on the results of this study, additional clinical studies are needed for patients with MGH.

## Ethics Statement

This study was carried out in accordance with the recommendations of the guide for the care and use of laboratory animals, published by the National Institutes of Health (USA). The protocol was approved by the Animal Care and Use Committee of Heilongjiang University of Chinese Medicine.

## Author Contributions

XLi designed the study and drafted the manuscript. YuW contributed to the analysis of TSP components. XLi, YZ, ZW, and XLei conducted the experiments, analyzed data, and interpreted results. PX, XF, NG, YS, BY, and YanW provided suggestions and material support. QW and HK designed the study, supervised the whole research work, and revised the manuscript. All authors approved the version of the manuscript.

## Conflict of Interest Statement

The authors declare that the research was conducted in the absence of any commercial or financial relationships that could be construed as a potential conflict of interest.

## Abbreviations

E2: Estradiol
FSH: follicle stimulating hormone
LH: luteinizing hormone
MGH: Mammary gland hyperplasia
NG: normal group
P: Progesterone
PRL: prolactin
T: testosterone
TSP: total saponins of Phytolaccae
